# Systemic Steroid Treatment for Severe Expanding Pneumococcal Pneumonia

**DOI:** 10.1155/2015/186302

**Published:** 2015-02-26

**Authors:** Eran Lavi, David Shoseyov, Natalia Simanovsky, Rebecca Brooks

**Affiliations:** ^1^Department of Pediatrics Mount Scopus, Hadassah-Hebrew University Medical Center, P.O. Box 24035, 91240 Jerusalem, Israel; ^2^Department of Medical Imaging, Hadassah-Hebrew University Medical Center, Mount Scopus, P.O. Box 24035, 91240 Jerusalem, Israel

## Abstract

The treatment of bacterial community-acquired pneumonia (CAP) is based on appropriate antibiotic therapy and supportive care such as intravenous fluids and supplemental oxygen. There is no available data regarding the use of steroids in CAP in children. We present an unusual case of a child with severe respiratory distress, on the brink of mechanical ventilation, due to a rapidly expanding pneumococcal pneumonia. The administration of systemic steroids resulted in a dramatic response with rapid improvement of clinical and radiological abnormalities followed by improvement of laboratory abnormalities. This case report should raise the awareness of the potential benefits of steroids in the treatment of severe pneumonia in children. Prospective randomized trials are needed to confirm the efficacy of steroids in this setting and to determine which patients would benefit most from this.

## 1. Introduction

Community-acquired pneumonia (CAP) is a significant cause of morbidity and mortality in childhood. Standard treatment for bacterial CAP consists of antibiotic therapy and supportive care, for example, intravenous fluids and supplemental oxygen. The role of systemic corticosteroids in the treatment of bacterial CAP has been reported in adults with conflicting results. We present a case of a child with severe expanding pneumococcal pneumonia that responded to systemic steroids.

## 2. Case Presentation

A 5-year-old, generally healthy girl, presented to the emergency room with a two-day history of fever, breathing difficulties, and cough. Upon admission she was dyspneic, febrile (38.2°C), and with a heart rate of 160 beats per minute. On auscultation crackles and reduced air entry to the left lung were diagnosed.

Laboratory results showed a normal white blood count with an elevated C reactive protein level (38.6 mg% (N < 0.5 mg%)) and mild respiratory acidosis. Blood cultures taken on admission were positive for* Streptococcus pneumonia* sensitive to Penicillin. The chest X-ray showed an extensive consolidation involving the entire left lung. The patient was admitted to the Pediatric Intensive Care Unit (PICU) where intravenous (IV) Penicillin treatment, IV fluids, and supplemental oxygen were initiated.

**Figure 1:**
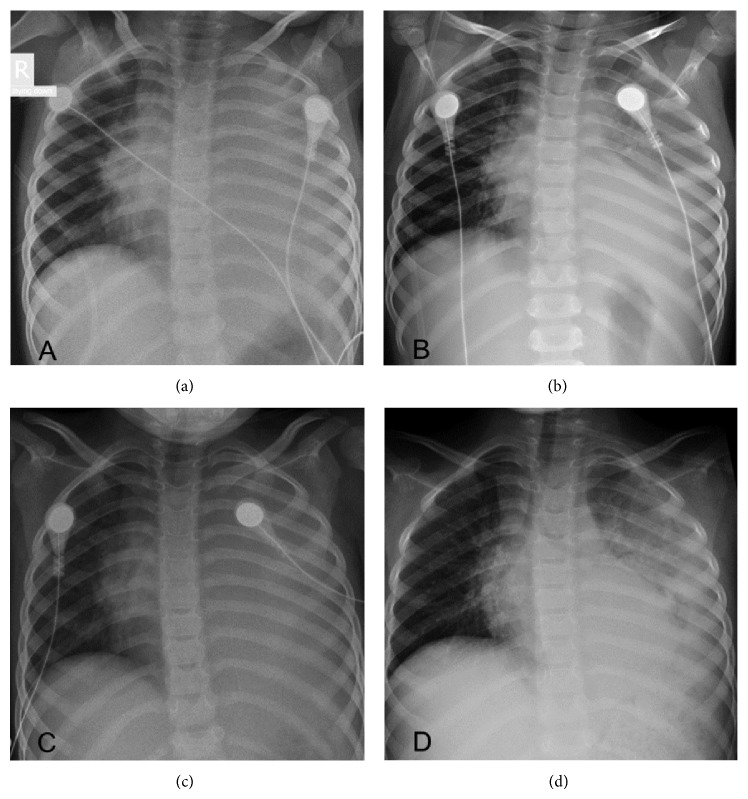
A 5-year-old patient with severe expanding pneumococcal pneumonia. (a) Note “white hemithorax” on the left with increased volume causing mediastinal shift to the right. (b) After a single course of left IV methylprednisolone. Significant improvement in the aeration of the left lung with residual consolidation above the diaphragm. (c) A clinical and radiologic deterioration. (d) Improved aeration of the left lung after a second course of IV steroids.

Despite the intensive treatment in the PICU, the patient's condition worsened with increased tachypnea and dyspnea requiring escalating amounts of supplemental oxygen. Both physical examination and a repeated chest X-ray showed pleural effusion. A pleural tap was performed and 200 mL of fluid drained. The antigen detection test and pleural fluid culture were positive for* Streptococcus pneumonia*. Despite fluid removal the patient continued to deteriorate. An additional chest X-ray showed severe expanding consolidation with a mediastinal shift to the right (Figure 1(a)). A chest computer tomography scan (CT) confirmed extensive parenchymal consolidation with only minimal pleural effusion.

A single dose of IV methylprednisolone (1 mg/kg) was administered 22 hours after admission to the PICU. Over the following hours the patient's condition improved substantially becoming less dyspneic with improved air entry to her left lung. Oxygen saturations normalized. A repeated chest X-ray 18 hours after steroid administration showed regression of the lung infiltrate with a normal positioned trachea (Figure 1(b)). However, 24 hours after initial IV steroid treatment she again deteriorated clinically. On the chest X-ray performed 32 hours after steroid treatment a recurrence of the mediastinal shift was detected (Figure 1(c)). A second dose of methylprednisolone was administered to which the patient responded again with the same remarkable response (Figure 1(d)). Steroid treatment was maintained at the dose of 1 mg/kg twice daily for 5 days. The patient continued to improve with no further complications and was discharged home in good condition after a total of 18 days of hospitalization.

It should be emphasized that during the entire period of hospitalization the only additional intervention to the standard treatment consisted of IV steroid therapy. Antibiotics remained unchanged.

## 3. Discussion

Pneumonia is an acute infection of the lung parenchyma, causing an inflammatory response which changes the balance between pro- and anti-inflammatory cytokines. IL-1*β* is associated with severity of infection, IL-6 reflects the severity of stress, and TNF-*α* may be a marker of pneumonia severity [1]. An excessive inflammatory response may lead to severe damage of the pulmonary tissue resulting in respiratory failure and/or septic shock.

Corticosteroids are powerful inhibitors of the inflammatory cascade, suppressing the expression of proinflammatory cytokines and thus potentially preventing an extended inflammatory response.

In infectious diseases the use of corticosteroids is known to reduce complications, such as hearing loss in* Haemophilus influenzae* bacterial meningitis [2], or the need for mechanical ventilation in cases of pneumonia caused by* Pneumocystis jiroveci* in HIV patients [3].

The role of steroids treatment in septic shock and sepsis was examined in several studies and remained controversial without clear evidence of improvement in mortality rate [4]. For this reason steroids are not generally recommended in sepsis management unless adrenal insufficiency is proven.

A small number of studies describe the use of steroids in adult patients with CAP. Meijvis et al. report on a cohort of patients with CAP randomly assigned to receive IV dexamethasone or placebo. Their results showed that length of hospital stay was significantly reduced in the dexamethasone group, whereas no difference was seen in mortality rate between the groups. The study did not include patients in the Intensive Care Unit [5]. Garcia-Vidal et al. showed reduced mortality among patients with CAP who received adjuvant steroid treatment compared with those who received antibiotics alone [6]. In a randomized double-blinded trial performed by Snijders et al., administration of prednisolone (40 mg) for one week did not improve the outcome of patients with CAP. Moreover in this study late failure of treatment was more common among patients treated with prednisolone than those in the placebo group [7].

The role of steroids for the treatment of CAP in the pediatric population has not been extensively studied and is limited mostly to case series. One study has shown clinical improvement after administration of methylprednisolone pulse therapy in 6 children with refractory* Mycoplasma pneumonia* [8].

In a multicenter retrospective study Weiss et al. reported that adjuvant corticosteroid therapy was associated among pediatric patients who received concomitant *β*-agonist therapy with a shorter hospital length of stay. However, in patients who did not receive *β*-agonists, systemic corticosteroids were associated with a longer length of stay and greater odds of readmission [9].

The case described here is of a child with severe respiratory distress, on the verge of mechanical ventilation, due to rapidly expanding pneumococcal pneumonia causing a mediastinal shift. The administration of systemic steroids resulted in a dramatic response. It should be emphasized that the patient was not in a septic shock and the deterioration was mainly respiratory. During hospitalization no adverse effects of steroids (hyperglycemia, superadded infection, and bleeding) were noted.

To our knowledge this is the first case report of a significant response to corticosteroids in a pediatric patient with pneumococcal CAP.

In summary, this case report should raise the awareness of the potential benefits of steroids in the treatment of severe pneumonia in children.

As previously shown in a number of studies, steroid treatment in different settings such as sepsis is not beneficial in all cases. The effect of steroids is probably multifactorial and depends on a variety of factors such as genetics, comorbidities, and the severity of the inflammatory response. However, as demonstrated in this case, steroid treatment may significantly reduce mortality in certain cases, yet our findings cannot be generalized to nonbacteremic pneumococcal pneumonia and prospective randomized trials are needed to determine which patients would benefit most from this treatment in both the adult and pediatric population.
